# Relaxation incisions and tensile strength in the abdominal wall of pigs ^1^

**DOI:** 10.1590/s0102-865020190060000008

**Published:** 2019-08-19

**Authors:** Aline Ribeiro Pedroso, Renato Miranda de Melo, Enio Chaves de Oliveira

**Affiliations:** I Fellow Master degree, Postgraduate Program in Health Sciences , Faculdade de Medicina , Universidade Federal de Goiás (FM-UFG), Goiania - GO , Brazil . Design of the study, technical procedures, acquisition and interpretation of data, manuscript writing.; II PhD, Associate Professor, Division of Operative Technique and Experimental Surgery , FM - UFG , Goiania - GO , Brazil . Conception, design, intellectual and scientific content of the study; technical procedures; critical revision.; III PhD, Associate Professor, Postgraduate Program in Health Sciences, Department of Surgery , FM - UFG , Goiania - GO , Brazil . Technical procedures, intellectual and scientific content of the study, critical revision.

**Keywords:** Incisional Hernia, Tensile Strength, Abdominal Wall, Swine

## Abstract

**Purpose:**

To analyze the resistance to medial traction of abdominal wall muscles, before and after performing relaxing incisions.

**Methods:**

Seventeen live pigs were used. After a median laparotomy, the handles were made in the rectus abdominis muscles (RAM) to fit the dynamometer. Step 1 (control phase): tensile strength measured without performing relaxant incisions. Step 2: A curvilinear relaxant incision was made on the anterior blade of the right RAM sheath and then the tensile strength was measured by the edge of the wound. The same procedure was adopted after incision of the left posterior blade. Step 3: Relaxing incisions were made in the right posterior and left anterior blade, so that both sides were left with a relaxing incision on both blades. Measurements of resistance were performed.

**Results:**

There was no statistically significant difference between the sides. On the right and left side, all treatments reduced the tensile strength when compared to each other and to the control. There was a reduction of 12% and 9.8% after incision of the anterior and posterior blade, respectively.

**Conclusion:**

Relaxing incisions reduced tensile strength in the ventral abdominal wall.

## Introduction

Laparotomy is considered a routine procedure in medical practice, with the median incision being the main access route to the abdominal cavity, offering wide visceral exposure. This incision, if performed in the alba line, causes little blood loss; however, in the supra-umbilical region, there is a greater incidence of eviscerations and eventrations, since it is a place subject to greater tensions ^1 - 4^ .

The abdominal wall suture should have a reduced rate of complications, such as wound dehiscence and incisional hernia (IH) formation. Excessive stress at the edges of the defect after repair is considered a major cause for the development of such complications ^5^ . Other factors are also associated with the development of IH, such as obesity, old age, malnutrition, technical error during repair, infection, ascites, postoperative hematoma, pregnancy, and other conditions that increase abdominal wall tension or compromise healing at the incision site ^3 , 6^ .

The search for adequate solutions for the lasting correction of hernias is frequent among surgeons, because, however small the defect, there will always be tension in the repair of the surgical wound. Thus, the reduction of this tension in the suture line is one of the objectives of the various techniques available for abdominal wall reconstruction ^7 - 10^ .

The use of synthetic mesh is very common in IH repair; however, some complications are attributed to the use of this material, such as the development of seroma, infection and fistula ^11 , 12^ . Another treatment option is parietal closure after relaxing incisions, which may or may not include separation of the lateral abdominal muscles ^13^ .

In order to avoid rejections, infections and foreign body reactions to biomaterials, the techniques that use the patient’s own tissues are an excellent treatment option. In this regard, Alcino Lázaro da Silva proposed a technique for the repair of IH, bilateral transitional peritoneo-aponeurotic transposition (TRANSPALB) or transposition with the hernial sac (TSH), where the patient’s hernial sac is used for the repair of the defect, after performing relaxing incisions in the sheath of the rectus abdominis muscles ^8 , 14^ .

The objective of this study was to compare the resistance to medial traction of the abdominal wall muscles and their aponeurotic components in live pigs before and after relaxing incisions on the anterior and posterior blades of the rectus abdominis sheath.

## Methods

The present study was carried out respecting the ethical principles of animal experimentation, according to the rules of the National Council for the Control of Animal Experimentation (CONCEA), Law N ^o^ 11.794 of October 8, 2008 (Lei Arouca) and Decree N ^o^ 6.899 of July 15, 2009. The Ethics Committee on the Use of Animals of the Pontifícia Universidade Católica de Goiás (CEUA/PUC-GO) approved the study under protocol N ^o^ 1745220817.

This is an experimental, quantitative and cross-sectional study that was conducted in the Laboratory of Surgical Technique of the Department of Medicine of the Pontifícia Universidade Católica de Goiás (PUC-GO), with live *Large White* pigs, after they had been used for the regular practical classes of Operative Technique of the students of the Medicine course.

Sampling was based on the literature, based on similar studies, which used 20 human cadavers. In this study, the initial sample consisted of 20 animals. However, the final sample of 17 female pigs with a body weight of 15 kg was obtained from the exclusion criteria. Animals with pre-existing lesion, defect or trauma of the abdominal wall were excluded, thus avoiding the possibility of alteration of the measured variables.

Initially, the animals were submitted to the general anesthesia protocol standardized for the species, under the care of the veterinarian responsible. This included the use of pre-anesthetic medication, induction and maintenance of anesthesia. The intramuscular (IM) association of morphine sulfate (0.1-0.2 mg/kg, Cristália, Itapira/SP, Brasil), ketamine hydrochloride 10% (5.0 mg/kg, Syntec, Santana de Parnaíba/SP, Brasil), midazolam (5.0 mg/kg, Cristália, Itapira/SP, Brasil) and xylazine hydrochloride (1.0 mg/kg, Konig, Mairinque/SP, Brasil) was used as pre-anesthetic medication. The marginal ear vein was punctured for administration of crystalloid solution (Lactated Ringer) and as a route for anesthetic induction. For this purpose, ketamine hydrochloride 10% (1.0-1.5 ml/animal) was used.

The animals were submitted to endotracheal intubation with a cannula of 5.0 to 5.5 mm in diameter. Maintenance occurred through the use of an inhalation anesthetic (isoflurane, BioChimico, Anápolis/GO, Brasil) with continuous oxygen flow.

**Figure 1:**
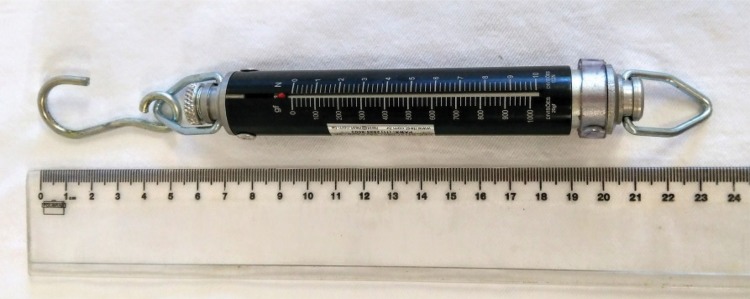
Crown AT portable analog dynamometer.

To monitor heart rate and oxygen saturation in the peripheral blood, we used a portable pulse oximeter, Model 1000 (J.G. Moriya, São Paulo/SP, Brasil). To measure the resistance to the medial traction offered by the muscle-aponeurotic joint, an analog dynamometer Crown AT was used (Oswaldo Filizola, São Paulo/SP, Brasil) with capacity up to 1kgf (kilogram-force) or 1000 gf (gram force), which was revised and calibrated after each measurement ( Fig. 1 ).

**Figure 2:**
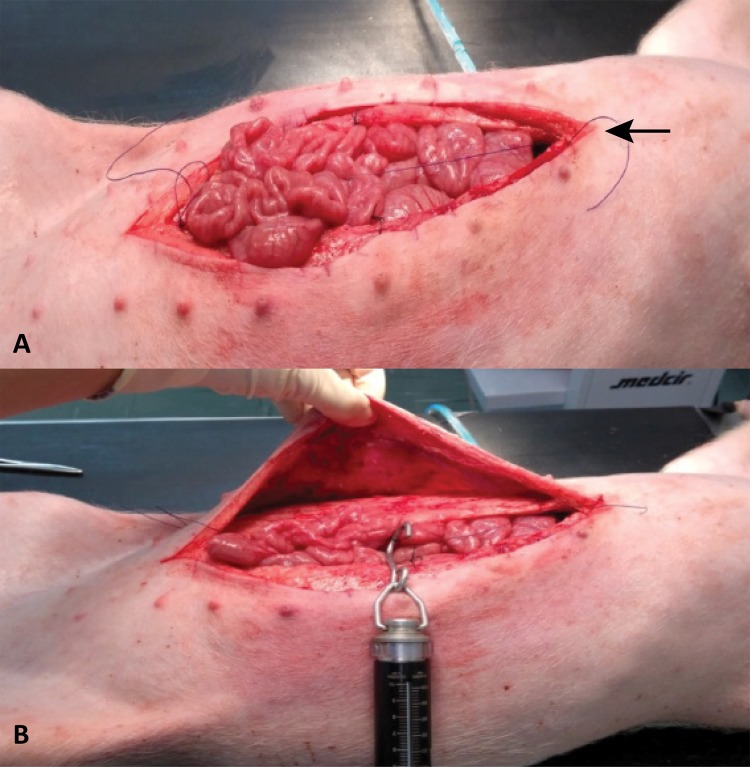
– A. Demarcation of the midline ( *arrow* ); B . Location of the handles for the dynamometer.

Initially, a median and xiphobic laparotomy was performed, bypassing the umbilical scar and exposing the sheath of the rectus abdominis muscle (RAM). After opening the abdominal cavity, the midline was demarcated with a single point of nylon 3.0 (Technofio, Goiânia/GO, Brasil). In the sequence, handles with cotton yarn 2.0 (Technofio, Goiânia/GO, Brasil) were made in the rectus abdominis muscles to fit the hook of the dynamometer ( Fig. 2 ). These handles, equidistant between the xiphoid process and the pube, were made on the right and left side of the incision, encompassing all of the muscle-aponeurotic components of the wall. The measurement of resistance to medial traction was performed in three stages.

In the first stage (control phase), the tensile strength was measured without performing relaxing incisions, pulling the dynamometer medially at 3.0 cm from the midline, on the right and left side of the laparotomic wound. Each animal was self-control.

**Figure 3:**
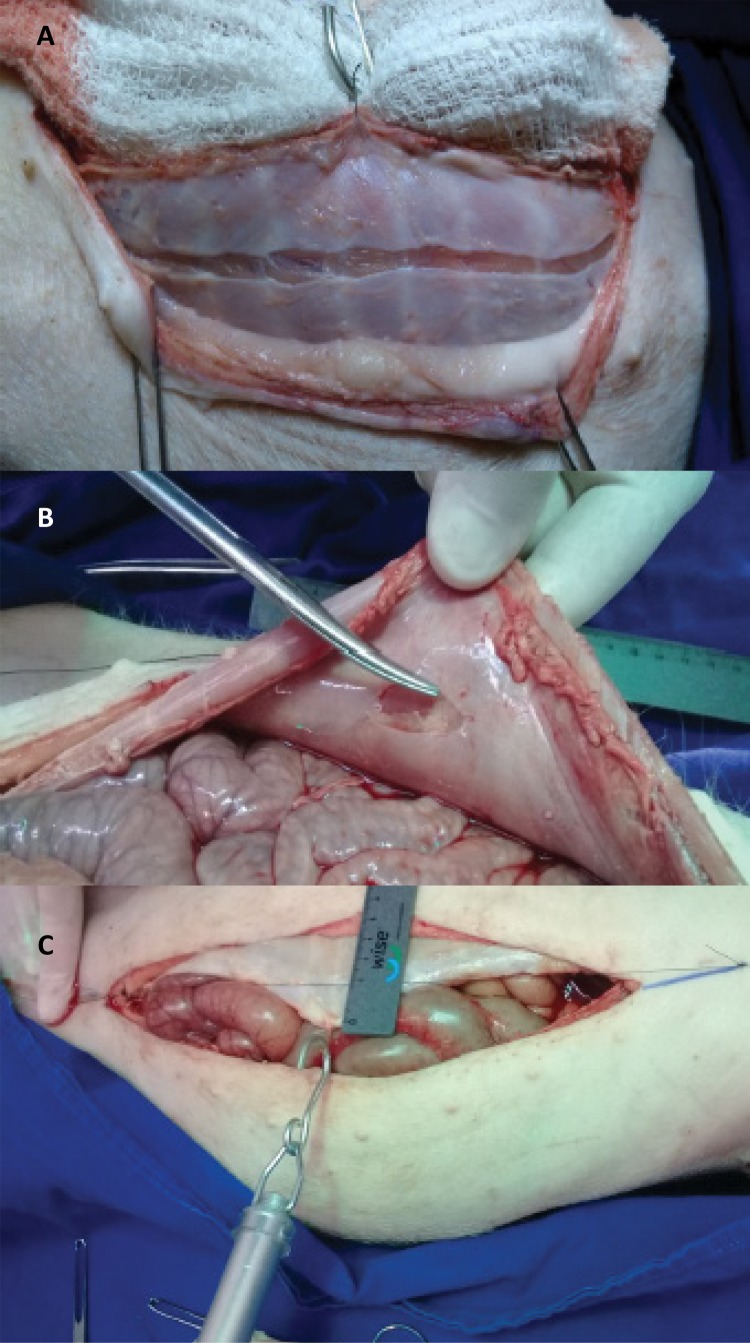
Initiating a relaxing incision on anterior (A) and posterior (B) blades of the rectus sheath. Tensile strength measurement 3.0 cm beyond the midline (C) .

In the second stage, a curvilinear relaxant incision was made in the anterior blade of the right rectus sheath, about 2.0 cm from the edge of the wound and along its entire length. The medial traction resistance was measured by the edge of the wound, exceeding the midline by 3.0 cm. The same procedure was adopted after incision of the posterior blade of the left rectus sheath ( Fig. 3 ).

In the third step, relaxant incisions with the same extension of the anterior ones were made in the posterior blade of the right rectum muscle sheath and in the anterior blade of the left sheath, so that both sides, right and left, had a relaxing incision on both blades (anterior + posterior). Then, the tensile strength measurement was performed with the same criteria used previously ( Fig. 3 ).

Three measurements of traction were performed in each animal, for each parameter analyzed: right control (RC), right anterior incision (RA), anterior + posterior right incision (A+PR), left control (LC), left posterior incision (LP) and anterior + posterior left incision (A+PL).

At the end of the procedure, the animals were submitted to euthanasia, using sodium thiopental 1g at the dosage of 33.0 mg/kg and potassium chloride 19.1%, 20.0 ml per animal intravenously (IV).

### Statistic

The data were analyzed by the Statistical Package for Social Sciences (SPSS, New York, USA) version 23.0, adopting a statistical significance level of 5% (p <0.05). Inferential analysis was performed with mean, standard deviation and confidence interval (95%). The normal distribution of the data was verified using the Shapiro-Wilk test (p> 0.05). The T-Paired test was applied to compare the means of the variables between the right and left sides. For comparison of the means of each side, the analysis of variance test (ANOVA) of repeated measures with the post hoc of Bonferroni was used.

## Results

**Figure 4:**
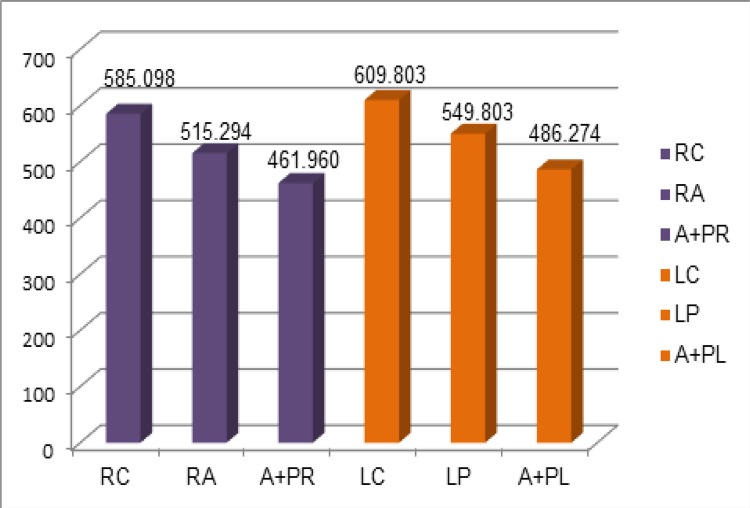
Average, in gF (grams-force), obtained from the realization of three strength measurements for each variable. RC: right control; RA: right anterior incision; A+PR: anterior + posterior right incision; LC: left control; LP: left posterior incision; A+PL: anterior + posterior left incision.

The averages of the values obtained for each variable are shown in Figure 4 .

The T-Paired test compared the averages between the right and left sides before and after the relaxant incisions. There was no statistically significant difference (p> 0.05) between them ( Table 1 ).

**Table 1:** Pairwise comparison between right and left sides for each variable, presenting mean discrimination and confidence interval (gF).

		Average	Standard deviation	Confidence interval (95%)		p*

				Inferior limit	Upper limit	
Stage 1	Right: Control ^1^	585.098	88.271	539.713	630.483	0.360
	Left: Control ^1^	609.803	106.591	555.000	664.608	
Stage 2	Right: Anterior ^2^	515.294	72.639	477.946	552.642	0.240
	Left: Posterior ^3^	549.803	148.419	473.494	626.114	
Stage 3	Right: Anterior+Posterior ^4^	461.960	69.112	426.427	497.495	0.371
	Left: Anterior+Posterior ^4^	486.274	111.964	486.2745	111.96405	

*T-Pareado test, considering 95% confidence with p ≤ 0.05.

1: before the relaxant incisions.

2: incision made in the right anterior blade of the rectus sheath.

3: Incision made in the left posterior blade of the rectus sheath.

4: Incision in both blades of the rectus sheath.

The averages of the right side of the laparotomic incision obtained in the three steps were compared with each other, using the Anova test with post hoc Bonferroni. It was observed that both the anterior and anterior+posterior incisions had a reduction in the tensile strength when compared to the control, as well as the anterior+posterior incision when compared to the anterior incision ( Table 2 ).

**Table 2:** Comparison of the right sheath incisions in the control, anterior and anterior + posterior (gF).

Right	Average	Standard deviation	Confidence interval (95%)		p*

			Inferior limit	Upper limit	
Control (A) ^1,3^	585.098	88.271	539.713	630.483	< 0.001
Anterior (B) ^1,2^	515.294	72.639	477.946	552.642	
Anterior+ Posterior (C) ^2,3^	461.961	69.112	426.427	497.495	

*P: Anova test of repeated measurements.

1 – P= <0,001; 2 – P= <0,001; 3 – P=0,001 (Bonferroni post hoc – paired comparison).

A: before the relaxant incisions.

B: incision made in the right anterior blade of the rectus sheath.

C: incision in both blades of the right rectus sheath.

**Figure 5:**
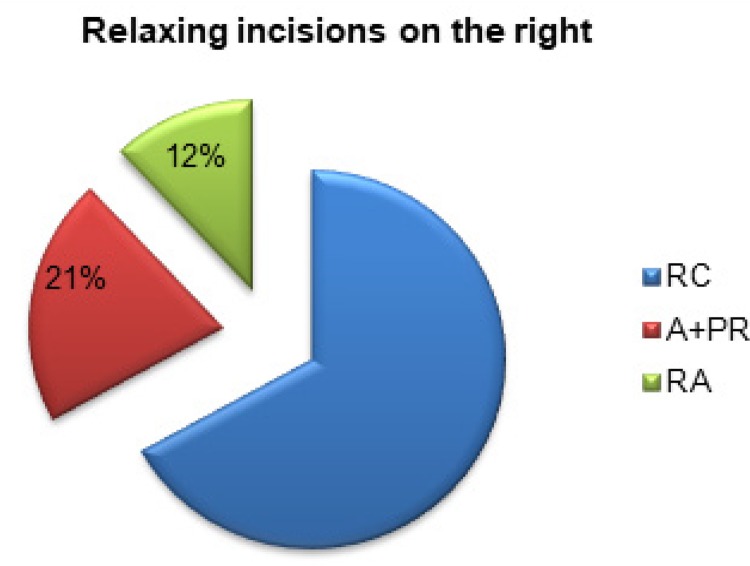
Percentage reduction in tensile strength after relaxation incisions to the right of the abdomen.

There was a reduction of 12% (69.804 gF) in traction force after incision of the right anterior blade of the RAM sheath and a 21% (123.137 gF) reduction after a relaxing incision in both blades of the right rectum sheath, anterior+posterior ( Fig. 5 ).

The averages of the left side of the laparotomic incision were also compared by the Anova test with post hoc Bonferroni, obtained in the three phases. It can be observed a reduction of the tensile strength in the ventral abdominal wall after the relaxation incisions; both the posterior and anterior+posterior incisions showed a reduction of this tensile strength when compared to the control, as well as the anterior + posterior incision when compared to the posterior incision ( Table 3 ).

**Table 3:** Comparison of the left sheath incisions in the control, posterior and anterior + posterior (gF).

Left	Average	Standard deviation	Confidence interval (95%)		p*

			Inferior limit	Upper limit	
Control (A) ^1,2^	609,803	106,591	555,000	664,608	< 0,001
Posterior (B) ^1,3^	549,803	148,419	473,494	626,114	
Anterior+ Posterior (C) ^2,3^	486,274	111,964	428,708	543,841	

*P: Anova test of repeated measurements.

1 – P= 0,001; 2- P= <0,001; 3 – P= 0,001 (Bonferroni post hoc – paired comparison).

A: before the relaxant incisions.

B: Incision made in the left posterior blade of the rectus sheath.

C: incision in both blades of the left rectus sheath.

**Figure 6:**
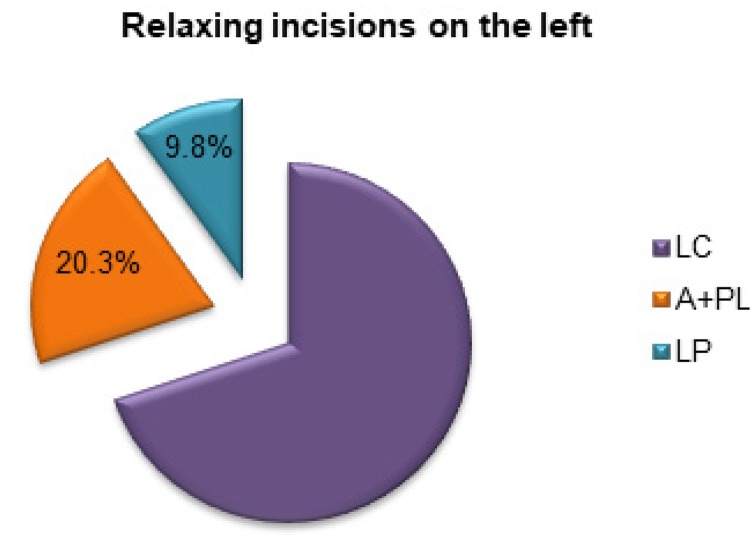
Percentage of reduction of tensile strength after performing relaxing incisions to the left of the abdomen.

After the incision of the left posterior blade of the sheath, there was a reduction of 9.8% (60 gF) in the tensile force and a reduction of 20.3% (123.529 gF) after incision of both blades of the left rectus sheath ( Fig. 6 ).

## Discussion

The reconstruction of the abdominal wall after surgical procedure, maintaining its physiological characteristics, becomes a challenge for each new patient. Regardless of the technique used, the repair of abdominal wall hernias has the objective of closing the defect without tension, with or without the use of prosthetic material, restoring the abdominal wall physiology, providing the patient with a quality of life, with a lasting and aesthetic ^15^ result.

The use of pig as an experimental model was considered effective for the objectives proposed in this study. Among the animals used as experimental models, the pig is the closest to the anatomical and functional characteristics of humans. Thanks to this anatomical and physiological similarity, the use of pig as a model for hernia repair is frequent ^16 , 17^ . Some authors have concluded that the muscular anatomy of the abdominal wall of pigs in the midline is very similar to that of humans, as well as in relation to blood supply and scarring ^18 , 17^ .

In the present case, unlike the Amorim study ^3^ , there was a normal distribution of collected values, which was determined by the Shapiro-Wilk test. This is one of the tests most used to test the normality of the data, and it is indicated, especially, in the case of small samples ^19^ . The model used in the present study allowed the construction of a paired sample, that is, the same animal provided the data before and after the intervention. Therefore, the paired t-test was used to compare the results of the right and left sides for each variable. Obtaining paired data, that is, related data, also allowed the use of ANOVA test of repeated measures to compare the means of the different treatments in the same group.

In the technique described by Nahas and Ferreira ^3 - 5 , 20^ , fresh human corpses were used, which had the alba line removed to promote a defect in the muscle-aponeurotic plane. The tensile strength measurement was also performed with an analog dynamometer. In the present study, no defect was observed in the abdominal wall components, as suggested by the previous studies; in this way, the RAM was drawn 3.0 cm beyond the midline in order to obtain a numerical value to apply the statistical tests.

The dynamometer adopted for the measurement of tensile strength is an analog model, composed of a spring that stretched as a force applied. The maximum supported capacity is 1kgF (one kilogram-force) or 1000gF (thousand grams-force), the scale being divided every 20gF (twenty grams-force). This dynamometer model has been used successfully in studies to evaluate the tensile strength in the abdominal wall of human cadavers ^3 - 5^ .

In other works, the traction values were used to calculate the traction coefficient, whose result is obtained by the traction value measured by the dynamometer (Kgf or gF) divided by the distance from the aponeurotic point to the midline (cm). In the present study, since the distance was standardized at 3.0 cm beyond the midline, it was not necessary to use this formula to calculate the coefficient of traction.

Because they are live animals, one can observe the influence of certain physiological processes at the moment of data collection, such as the accumulation of gases in the intestine, generating a distension of the same, which made difficult the measure of traction in some animals. Such facts can be confirmed by Levine and Karp ^21^ , who claim that in living beings, the tension in the abdominal wall can suffer influenced abdominal content, which generates a centrifugal force, pressing the components of the wall; and also by the contraction of the abdominal muscles, which generate a lateral force vector.

In the present study, there was no interference of post-mortem changes in the sample, such as rigor mortis, which occurs when the energy sources in the muscle fibers become depleted, and these lose their extensibility, becoming rigid ^22 , 23^ . The onset and duration of rigor mortis in pigs is quite variable, and can occur from one to three hours after death, lasting around 6 hours ^24^ . In humans, rigor mortis begins approximately 2 to 6 hours after death and lasts about 36 hours ^22^ .

The traction evaluation was performed at only one point in the supra-umbilical region on both sides of the laparotomic wound, encompassing the entire muscle-aponeurotic component, unlike other studies ^3 - 5^ , in which the evaluation of resistance to traction was applied at different points of the surgical wound, both in the supra and infra-umbilical regions. These studies demonstrated that there was no statistically significant difference between the different points tested in the same aponeurosis; this is why we chose only one evaluation point.

Comparing the right and left sides of the laparotomy, both in the control phase and after the incisions of the anterior and posterior laminae of the RAM sheath, no statistically significant difference was observed, that is, the mean traction of the muscle-aponeurotic components was very close in both sides, demonstrating similarity between the components of the abdominal wall both to the right and to the left of the laparotomy.

On the right side we can observe a significant reduction in the means of traction between the control and the anterior incision. After the relaxation incision was performed on the anterior leaf of the right sheath of the RAM, there was a reduction of 12% (69.804 gF) in the traction measure, in relation to the control measure. There was also a significant reduction between the incision in the left leaflet’s posterior blade in relation to the control mean; being this reduction of 9.8% (60 gF). In this way, it can be verified that the incision of the anterior blade of the sheath generated a greater reduction of the resistance to the traction when compared with the incision of the posterior blade.

These results are consistent with other studies, which demonstrated that in the epigastric region, the anterior blade of the rectum sheath resisted a greater horizontal traction load in relation to the posterior blade. They concluded that in both horizontal and vertical direction, the stability (resistance capacity) was significantly higher in the anterior sheath, above the arcuate line, than in the posterior ^25^ . It is also in line with the results of Amorim *et al* . ^3^ , in which it was found that there was no difference in the medial tensile strength at the different levels studied along the anterior and posterior sheath of the rectus muscle; however, when comparing the anterior and posterior sheath, it was noticed that the resistance to the medial traction was significantly greater in the anterior layer. It was concluded from these studies that the anterior sheath is more resistant to traction in relation to the posterior sheath; so, the reduction in tensile strength was more significant after incision of this blade.

According to Amorim *et al* . ^3^ , this difference in tension between the layers (anterior and posterior) can be explained by the amount and arrangement of the lateral fibers of the rectus muscle. In the anterior sheath of the rectus muscle, there are bundles of oblique fibril, whereas in the posterior sheath prevail the bundles of transverse fibril. These collagen fibers, present in the fascia and muscles, are important stabilizing components of the abdominal wall, whose orientation influences the mechanical characteristics of the tissue, being determinant for the resistance of these structures ^26 , 27^ .

Fachinelli and Trindade ^28^ , in their study to evaluate the amount of collagen in patients with hernia in the anterior wall, demonstrated that the amount of total collagen, type I and type III, was lower in patients with hernia than in cadavers. In this way, they could conclude that patients with ventral hernia (umbilical, epigastric and incisional) had a lower amount of collagen than the control group (without hernia).

From these studies it was concluded that the RAM sheath is a tissue that has a certain amount of collagen fibers in its composition, which gives it some resistance; that is, by practicing a relaxation incision in this structure, there is a reduction in the tensile strength of the muscle-aponeurotic components, facilitating the correction of the defect.

Axer *et al.* ^26^ also demonstrated that there was no statistically significant difference between the right and left sides in relation to the diameter or distribution of the fibrillary bundles. It can be said then that these findings justify the results found in the present study, since there was no statistically significant difference between the left and right sides, being a fabric with the same characteristics on both sides.

Another study also demonstrated that the relaxant incisions performed on the anterior rectus sheath significantly reduced the incidence of wound dehiscence and incisional hernia, since this technique increased the elasticity of the wound by reducing the tension in the suture line. In addition, the intensity of postoperative pain in these patients with relaxation incisions was lower than in the control group ^29^ .

Silveira *et al* . ^4^ , also studied the tension along the entire anterior sheath of the rectus muscle, and found no statistically significant difference between the various points studied, which means that there was no difference in the resistance to the medial traction; therefore, there is no place in the previous sheath that can be closed with less tension. For this reason, it is important to study techniques that seek to minimize tension for the correction of major defects and adequate closure of the abdominal cavity.

As future perspectives, new studies may be developed based on this methodology, using live experimental models, in order to verify the effectiveness of the methods performed here.

## Conclusions

After assessing the tensile strength of the abdominal-wall muscle aponeurotic components, before and after the relaxation incisions practiced, it was concluded that these reduced the tensile strength in the abdominal wall, being more significant after the incision of the anterior leaf of the sheath of the abdomen muscle, compared to the posterior blade. In relation to the experimental model used, the live pig was effective to the objectives proposed in this study.

## Footnotes

1 Research performed at the Laboratory of Surgical Technique, School of Medical, Pharmaceutical and Biomedical Sciences of Pontifícia Universidade Católica de Goiás, (PUC-GO), Goiania-GO, Brazil. Part of Master degree thesis, Postgraduate Program in Health Sciences, UFG. Tutor: Prof. Enio Chaves de Oliveira.
